# High-Coverage ITS Primers for the DNA-Based Identification of Ascomycetes and Basidiomycetes in Environmental Samples

**DOI:** 10.1371/journal.pone.0040863

**Published:** 2012-07-12

**Authors:** Hirokazu Toju, Akifumi S. Tanabe, Satoshi Yamamoto, Hirotoshi Sato

**Affiliations:** 1 The Hakubi Center for Advanced Research, Kyoto University, Sakyo, Kyoto, Japan; 2 Division of Biological Science, Graduate School of Science, Kyoto University, Sakyo, Kyoto, Japan; 3 Kansai Research Center, Forestry and Forest Products Research Institute, Nagaikyutaro-68, Momoyama, Fushimi, Kyoto, Japan; Université Paris-Sud, France

## Abstract

The kingdom Fungi is estimated to include 1.5 million or more species, playing key roles as decomposers, mutualists, and parasites in every biome on the earth. To comprehensively understand the diversity and ecology of this huge kingdom, DNA barcoding targeting the internal transcribed spacer (ITS) region of the nuclear ribosomal repeat has been regarded as a prerequisite procedure. By extensively surveying ITS sequences in public databases, we designed new ITS primers with improved coverage across diverse taxonomic groups of fungi compared to existing primers. An *in silico* analysis based on public sequence databases indicated that the newly designed primers matched 99% of ascomycete and basidiomycete ITS taxa (species, subspecies or varieties), causing little taxonomic bias toward either fungal group. Two of the newly designed primers could inhibit the amplification of plant sequences and would enable the selective investigation of fungal communities in mycorrhizal associations, soil, and other types of environmental samples. Optimal PCR conditions for the primers were explored in an *in vitro* investigation. The new primers developed in this study will provide a basis for ecological studies on the diversity and community structures of fungi in the era of massive DNA sequencing.

## Introduction

Fungi constitute a highly species-rich kingdom, embracing *c*. 100,000 described and potentially 1.5–5.1 million undescribed species [1]–[4] in environments such as forest soil [5], [6], phyllosphere [7]–[9], aquatic ecosystems [10], [11], and soils of the polar region [12]. Fungi are important components of ecosystems, acting, among other things, as decomposers [13], mutualists of plants [5], [6], and parasites of various organisms [14], [15]. They are utilized by humans in terms of applications in agriculture [16], [17], pharmacology [18], [19], the food industry [20], and environmental technologies [21]. Thus, exploration of fungal diversity is crucial not only for ecosystem and community ecology but also to provide invaluable resources for various fields of applied microbiology.

**Figure 1:**
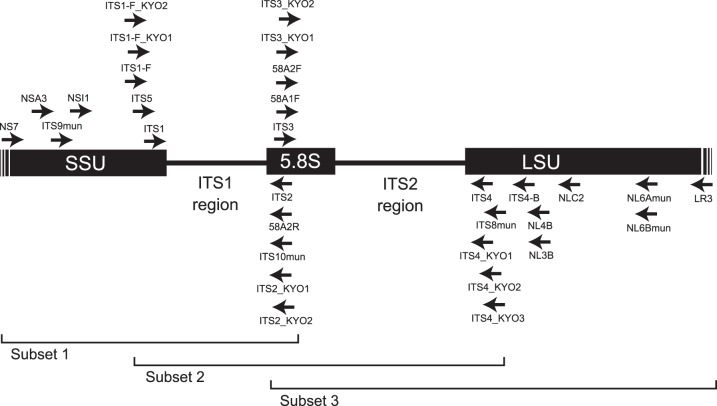
Map of nuclear ribosomal RNA genes and their ITS regions. Positions of forward (right-pointing arrow) and reverse (left-pointing arrow) primers are shown on the map of ITS regions and the surrounding ribosomal RNA genes. The ranges covered by the respective subset databases (see text) are also indicated.

In cataloging diverse fungal species and thereby constructing reference databases of fungal diversity, “DNA barcoding” based on the nucleotide sequence information of a target gene region can be highly efficient, potentially enabling the rapid and accurate identification of fungal specimens [22]–[25]. DNA barcoding is a method of identifying unknown samples by means of known classifications [26]. Generally, short nucleotide sequences in one to three loci are used as DNA barcoding markers to catalog and identify a taxonomic group [26]–[31]. The potential importance of DNA barcoding in mycology and ecology is enormous. Through identifying non-culturable or inconspicuous microscopic fungi lacking clear morphological characters (e.g., fruiting bodies), standardized DNA sequence information is expected to dramatically improve the efficiency of ecological and microbiological investigations [22], [23]. Likewise, DNA barcoding studies have recently shown the existence of many “cryptic” fungal species, which cannot be distinguished morphologically [32], [33], stressing that molecular information is indispensable for the identification and description of fungal species. Moreover, technological advancement in next-generation sequencing has opened the era of high-throughput DNA barcoding, fueling metagenomic studies of fungal communities in various environmental samples, such as mycorrhizae, leaves, and soil [9], [34]–[38]. To date, more than 90,000 fungal internal transcribed spacer (ITS) region sequences (Fig. 1), which is one of the most widely used barcoding regions of fungi [22]–[25], have been deposited in public databases [23]. The clustering of the molecular data further revealed that the 90,000 sequences represented *c*. 17,000 fungal taxa at a cutoff of 93% molecular identity [23]. These facts suggest the existence of enormous resources for molecular identification of fungal species based on ITS sequences, but that ITS sequences of only a small fraction of described and undescribed fungal species have been deposited in public DNA databases. Thus, the ITS sequences of the vast majority of fungi remain to be revealed.

In DNA barcoding, the universality of PCR primers limits the efficiency of cataloging and identifying specimens, and the development and selection of high-coverage barcoding primers are regarded as crucial steps [27], [39]. In fact, many attempts have been made to design high-coverage primers for amplification of the fungal ITS region [40]–[43]. Several “classic” primers developed by White *et al.* [40] and Gardes & Bruns [41] are commonly used for ITS-based barcoding of a variety of fungal groups [44], promoting various taxonomic [33] and ecological [2], [45] studies. However, a recent study showed that some of these primers can mismatch with target fungal ITS sequences at non-negligible frequencies, potentially precluding the DNA barcoding of some subgroups of fungi or hampering the accurate description of fungal community structures [39]. For example, the classic primers ITS2 and ITS3, which were designed to amplify the ITS1 and ITS2 regions (Fig. 1), respectively, do not match with the sequences of many basidiomycete fungi (the phylum Basidiomycota), causing biased amplification toward ascomycetes (the phylum Ascomycota) and other fungal groups such as glomeromycetes (the phylum Glomeromycota) [39]. Likewise, the fungi-specific primer ITS1-F, which facilitates the selective amplification of fungal ITS sequences from mycorrhizal and other types of environmental samples [41], shows a high proportion of mismatches to ascomycete sequences, resulting in biased amplification of basidiomycete and other fungal sequences [39]. Such biases are especially unfavorable in metagenomic studies on fungal diversity and community structures because the coverage of used primers severely influences the reliability of the data produced by next-generation sequencing [39], [46]. Consequently, a need exists to develop new primers that can be used for DNA barcoding of fungi with less taxonomic bias.

By thoroughly analyzing the sequence database of the fungal ITS region, we developed herein new primers showing enhanced coverage across diverse fungal taxa. An *in silico* analysis of the coverage of designed primers based on the ecoPCR program [39] revealed that the newly designed primers covered 99% of fungal taxa at the species level. Furthermore, they could amplify the sequences of diverse Dikarya fungi (i.e., a subkingdom of fungi consisting of the two phyla Ascomycota and Basidiomycota) without significant taxonomic biases. Although the limited number of “non-Dikarya” database sequences precluded a thorough examination of the coverage across the entire fungal kingdom, a further inspection of the *in silico* results showed that the newly designed primers could also cover an ecologically important clade of mycorrhizal fungi, Glomeromycota. Taking into account the coverage of each primer, high-coverage primer sets were selected for DNA barcoding of ITS1, ITS2, and the entire ITS region. Furthermore, to determine the optimal PCR conditions for each primer set and evaluate variation in amplicon length, which may cause taxonomic bias in PCR and next-generation sequencing (e.g., 454 pyrosequencing), *in vitro* PCR with ascomycete and basidiomycete specimens was conducted. This study provides a series of improved barcoding primers to promote ecological and microbiological studies of the diversity and community structures of fungi in the era of massive DNA sequencing.

**Table 1:** Primers used in this study.

Category	Primer name	Position	Primer sequence, 5'−3'	Primer source study
SSU (forward)	NSA3	1514–1535	AAACTCTGTCGTGCTGGGGATA	Martin & Rygiewicz (2005)
	ITS9mun	1618–1635	TGTACACACCGCCCGTCG	Egger (1995)
	NSI1	1644–1663	GATTGAATGGCTTAGTGAGG	Martin & Rygiewicz (2005)
	ITS1-F	1723–1744	CTTGGTCATTTAGAGGAAGTAA	Gardes & Bruns (1993)
	ITS1-F_KYO1	1723–1744	CTHGGTCATTTAGAGGAASTAA	This study
	ITS1-F_KYO2	1733–1753	TAGAGGAAGTAAAAGTCGTAA	This study
	ITS5	1737–1758	GGAAGTAAAAGTCGTAACAAGG	White et al. (1990)
	ITS1	1761–1779	TCCGTAGGTGAACCTGCGG	White et al. (1990)
5.8S (reverse)	ITS2	2024–2043	GCTGCGTTCTTCATCGATGC	White et al. (1990)
	58A2R	2026–2042	CTGCGTTCTTCATCGAT	Martin & Rygiewicz (2005)
	ITS10mun	2026–2043	GCTGCGTTCTTCATCGAT	Egger (1995)
	ITS2_KYO1	2026–2042	CTRYGTTCTTCATCGDT	This study
	ITS2_KYO2	2029–2046	TTYRCTRCGTTCTTCATC	This study
5.8S (forward)	ITS3	2024–2043	GCATCGATGAAGAACGCAGC	White et al. (1990)
	58A1F	2024–2040	GCATCGATGAAGAACGC	Martin & Rygiewicz (2005)
	58A2F	2026–2042	ATCGATGAAGAACGCAG	Martin & Rygiewicz (2005)
	ITS3_KYO1	2026–2043	AHCGATGAAGAACRYAG	This study
	ITS3_KYO2	2029–2046	GATGAAGAACGYAGYRAA	This study
LSU (reverse)	ITS4	2390–2409	TCCTCCGCTTATTGATATGC	White et al. (1990)
	ITS4_KYO1	2390–2409	TCCTCCGCTTWTTGWTWTGC	This study
	ITS4_KYO2	2401–2418	RBTTTCTTTTCCTCCGCT	This study
	ITS8mun	2433–2450	CTTCACTCGCCGTTACTA	Egger (1995)
	ITS4_KYO3	2442–2459	CTBTTVCCKCTTCACTCG	This study
	ITS4-B	2526–2548	CAGGAGACTTGTACACGGTCCAG	Gardes & Bruns (1993)
	NLB4	2558–2577	GGATTCTCACCCTCTATGAC	Martin & Rygiewicz (2005)
	NLB3	2559–2577	GGATTCTCACCCTCTATGA	Martin & Rygiewicz (2005)
	NLC2	2628–2649	GAGCTGCATTCCCAAACAACTC	Martin & Rygiewicz (2005)
	NL6Amun	2767–2786	CAAGTGCTTCCCTTTCAACA	Egger (1995)
	NL6Bmun	2767–2786	CAAGCGTTTCCCTTTCAACA	Egger (1995)
Subset construction only	NS7	1403–1426	GAGGCAATAACAGGTCTGTGATGC	White et al. (1990)
	LR3	3029–3045	CCGTGTTTCAAGACGGG	Vilgalys & Gonzalez (1990)

Both primers whose coverage of fungal taxa was evaluated and those used only for subset construction are shown, together with their position in a reference ribosomal RNA sequence of *Serpula himantioides* (AM946630).

## Methods

### Primer Design

To design new ITS primers that amplify broad fungal taxa, we constructed databases of fungal ribosomal RNA gene sequences. Two local sequence databases were prepared by downloading sequences from the Nucleotide database of the National Center for Biotechnology Information (NCBI; http://www.ncbi.nlm.nih.gov/; accessed April 26, 2011). To construct a database of small subunit ribosomal RNA gene sequences, 134,928 sequences were downloaded using the taxonomic ID 4751 (fungi) and the query word ‘small subunit OR 18S’ and setting the range of sequence length from 200 to 5,000 bp. Likewise, to construct a database of 5.8S ribosomal RNA and large subunit RNA gene sequences, 26,616 sequences were downloaded using the taxonomic ID 4751 (fungi) and the query word ‘5.8S’ with the abovementioned range of sequence lengths. Note that we attempted to construct a specific database of large subunit ribosomal RNA gene sequences using the query word ‘large subunit OR 28S,’ but many downloaded sequences did not contain the 5′-end of the gene, which was necessary for designing reverse primers for the ITS2 region (Fig. 1). Since each database was composed of numerous sequences, which were difficult to align simultaneously, consensus sequences of closely related taxa were obtained using assembler programs. Assembly was first conducted by PCAP [47], which enabled parallelized rapid assembling, with a sequence identity setting of 92%. The contigs returned by PCAP were further assembled to generate consensus sequences of diverse fungal taxa using CAP3 [48], which facilitated accurate assembling, with a sequence identity setting of 80% and a max overhang percent length of 200 bp. Multiple alignments of the output consensus sequences were conducted using MAFFT v6.813b [49] with default options. High-coverage PCR primers targeting the ITS1 and ITS2 regions were designed at conserved nucleotide positions of the aligned consensus sequences.

**Table 2:** Seven ascomycete and seven basidiomycete fungi are shown with the accession numbers of their ITS sequences.

ID	Species	Family	Order	Phylum	GenBank accession
A1	*Podostroma cornu-damae* (Pat.) Boedijn	Hypocreaceae	Hypocreales	Ascomycota	AB509797
A2	*Leotia lubrica* (Scop.) Pers.: Fr.	Leotiaceae	Helotiales	Ascomycota	AB509686
A3	*Phillipsia domingensis* (Berk.) Berk.	Sarcoscyphaceae	Pezizales	Ascomycota	AB509610
A4	*Xylaria* sp.	Xylariaceae	Xylariales	Ascomycota	AB509642
A5	*Cordyceps nutans* Pat.	Cordycipitaceae	Hypocreales	Ascomycota	AB509505
A6	*Vibrissea truncorum* Fr.	Vibrisseaceae	Helotiales	Ascomycota	AB509599
A7	*Trichocoma paradoxa* Jungh.	Trichocomaceae	Eurotiales	Ascomycota	AB509823
B1	*Auricularia aff. auricula* (Hook.) Underw.	Auriculariaceae	Auriculariales	Basidiomycota	AB509633
B2	*Bjerkandera adusta* (Willd.: Fr.) Karst.	Meruliaceae	Polyporales	Basidiomycota	AB509484
B3	*Laccaria vinaceoavellanea* Hongo	Hydnangiaceae	Agaricales	Basidiomycota	AB509671
B4	*Geastrum mirabile* (Mont.) Fisch.	Geastraceae	Geastrales	Basidiomycota	AB509736
B5	*Boletus ornatipes* Peck	Boletaceae	Boletales	Basidiomycota	AB509727
B6	*Thelephora aurantiotincta* Corner	Thelephoraceae	Thelephorales	Basidiomycota	AB509809
B7	*Amanita farinosa* Schw.	Amanitaceae	Agaricales	Basidiomycota	AB509651

DNA was extracted from fruiting body specimens collected on Yakushima Island, Kagoshima Prefecture, Japan. The fruiting body specimens were deposited in Kyoto University Herbarium (KYO). See Kirk *et al.* [3] and NCBI Taxonomy (http://www.ncbi.nlm.nih.gov/guide/taxonomy/) for the taxonomy of the specimens.

### *In silico* PCR

The coverage of the designed ITS primers was evaluated and compared with those of existing fungal ITS primers developed in previous studies [40]–[43] based on *in silico* PCR using the ecoPCR v0.7.0 software [39]. Since the software requested sequence database files of focal taxonomic groups in the European Molecular Biology Laboratory (EMBL) database, fungal nucleotide sequence databases (STD, GSS, HTG, and WGS of release 107) were downloaded from the EMBL FTP site (ftp://ftp.ebi.ac.uk/pub/databases/embl/release/). Given that only a small proportion of sequences covered the entire part of the small subunit 5.8S and large subunit ribosomal RNA genes and the flanking ITS1 and ITS2 regions (Fig. 1), three subset databases covering either ITS1, ITS2, or the entire ITS region were constructed, as detailed in Bellemain *et al.* [39]. To create the first subset (subset 1), the ITS1 region was amplified *in silico* by extracting sequences matching both NS7 and ITS2 primers (Table 1, Fig. 1) from the initial fungal database. In this process, three mismatches to each primer were tolerated but sequences containing consecutive five or more ‘N’ were excluded, leaving a database containing 1,374 sequences. Likewise, subset 2 for the entire ITS region (ITS5-ITS4; 8,421 sequences) and subset 3 for the ITS2 region (ITS3-LR3; 3,217 sequences) were created.

Based on the three subset databases, the coverage of each ITS primer (Table 1) was evaluated *in silico*. In the *in silico* amplification, each target primer was paired with the forward or reverse primer that had been used in making the focal subset database, and from zero to three mismatches between each target primer and the template sequence (except mismatches at the two bases of the 3′-end of the primer) were allowed to simulate different PCR stringency conditions. Three mismatches between each nontarget primer and the template sequence were allowed in all cases to remove the effects of nontarget primers. Since each subset database included multiple sequences of the same fungal species, the coverage of PCR primers was calculated in terms of the proportion of “taxa” amplified *in silico*. In our subset databases, each sequence had its own taxonomic IDs in the NCBI taxonomy database, and the lowest taxonomic units (LTUs) of the subset/database sequences corresponded to species, subspecies, or varieties in the NCBI taxonomy. In total, 723 LTUs in subset 1, 3,474 in subset 2, and 1,869 in subset 3 were subjected to *in silico* PCR. Using the taxonomic information, the results of *in silico* PCR were evaluated independently for ascomycetes, basidiomycetes, and the remaining “non-Dikarya” fungi. Compared to ascomycete and basidiomycete fungi, “non-Dikarya” fungi represented only 7.6% (subset 1), 8.3% (subset 2) and 2.9% (subset 3) of the database sequences.

**Figure 2:**
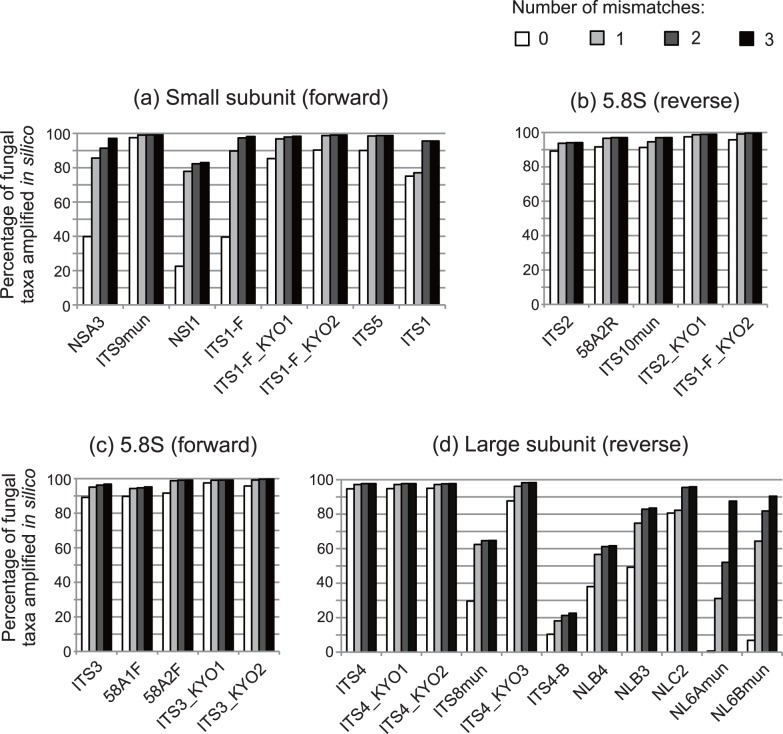
Coverage of fungal ITS primers. The percentage of fungal LTUs amplified by ecoPCR is shown for each primer. In each analysis, *in silico* amplification was conducted using both a target primer and the primer used in construction of the focal subset database; hence, the percentage represents the coverage of the target primer but not that of the primer pairs. From zero to three nucleotide mismatches between the target primer and database sequences were allowed in each analysis (except mismatches in the two bases at the 3′-end of the primer). (a) Forward primers for the small subunit ribosomal RNA gene evaluated with subset 1 (723 LTUs). (b) Reverse primers for the 5.8S ribosomal RNA gene evaluated with subset 2 (3,474 LTUs). (c) Forward primers for the 5.8S ribosomal RNA gene evaluated with subset 2 (3,474 LTUs). (d) Reverse primers for the large subunit ribosomal RNA gene evaluated with subset 3 (1,869 LTUs).

When selecting fungal PCR primers for DNA barcoding of environmental samples, both the coverage of primers across fungal taxa (species, subspecies, or varieties; see the above discussion of LTUs) but also the degree of “mismatches” to plant ITS sequences are important criteria for preventing the amplification of nontarget organismal sequences. Accordingly, we conducted *in silico* PCR of plant ITS sequences using fungal ITS primers (Table 1). From the EMBL FTP site (ftp://ftp.ebi.ac.uk/pub/databases/embl/release/), we downloaded plant nucleotide sequence databases (STD, GSS, HTG, and WGS of release 107) and used them to construct subsets 1–3, as detailed above. *In silico* PCR was conducted using the same settings as the fungal analysis; the results was are shown separately for Embryophyta (i.e., land plants) and the remaining “non-Embryophyta” plants.

On account of the coverage across fungal LTUs and the degree of mismatches to plant ITS sequences, candidate pairs of ITS primers for fungal DNA barcoding were selected for ITS1, ITS2, or the entire ITS region. The PCR fragment lengths amplified by these primer pairs were simulated using ecoPCR.

**Table 3:** Percentage of fungal taxa amplified *in silico*.

Category	Subset	Target primer name	Ascomycota	Basidiomycota	‘non-Dikarya’	Total
SSU (forward) *	Subset 1	NSA3	93.6	85.6	5.5	85.6
	(550 Ascomycota,	ITS9mun	99.6	98.3	94.5	99.0
	118 Basidiomycota	NSI1	88.0	43.2	50.9	77.9
	and 55	ITS1-F	92.2	80.5	85.5	89.8
	‘non-Dikarya’ LTUs)	ITS1-F_KYO1	98.4	94.9	85.5	96.8
		ITS1-F_KYO2	99.1	99.2	94.5	98.8
		ITS5	98.9	98.3	94.5	98.5
		ITS1	73.8	85.6	90.9	77.0
5.8S (reverse)†	Subset 2	ITS2	97.1	88.1	96.5	93.7
	(1,897 Ascomycota,	58A2R	97.0	97.2	91.3	96.6
	1,289 Basidiomycota	ITS10mun	97.0	91.6	91.3	94.6
	and 288	ITS2_KYO1	99.5	97.4	99.0	98.7
	‘non-Dikarya’ LTUs)	ITS2_KYO2	99.5	98.6	99.0	99.1
5.8S (forward)‡	Subset 2	ITS3	98.3	90.2	96.2	95.1
	(1,897 Ascomycota,	58A1F	98.5	90.1	85.8	94.3
	1,289 Basidiomycota	58A2F	99.3	98.4	97.6	98.8
	and 288	ITS3_KYO1	99.3	98.7	99.0	99.1
	‘non-Dikarya’ LTUs)	ITS3_KYO2	99.5	98.7	99.0	99.2
LSU (reverse)§	Subset 3	ITS4	97.6	96.9	92.6	97.2
	(1,102 Ascomycota,	ITS4_KYO1	97.6	96.9	92.6	97.2
	713 Basidiomycota	ITS4_KYO2	97.9	96.6	90.7	97.2
	and 54	ITS8mun	41.7	93.8	72.2	62.4
	‘non-Dikarya’ LTUs)	ITS4_KYO3	96.6	96.9	77.8	96.1
		ITS4-B	0.0	47.5	0.0	18.1
		NLB4	62.1	52.6	0.0	56.7
		NLB3	64.6	90.5	75.9	74.8
		NLC2	74.1	96.9	53.7	82.2
		NL6Amun	52.4	0.7	0.0	31.1
		NL6Bmun	51.0	84.7	68.5	64.4

One nucleotide mismatch between each target primer and the database sequence was allowed in ecoPCR, except for the two bases at the 3′-end of the primer.

* ITS2, used to construct subset 1, was used as a reverse primer in ecoPCR.

† ITS5, used to construct subset 2, was used as a forward primer in ecoPCR.

‡ ITS4, used to construct subset 2, was used as a reverse primer in ecoPCR.

§ ITS3, used to construct subset 3, was used as a forward primer in ecoPCR.

### In vitro Selection of Annealing Temperatures

Optimal annealing temperatures for the selected fungal ITS primer pairs were explored *in vitro*. Three ascomycete and four basidiomycete fungal species collected on Yakushima Island, Kagoshima Prefecture, Japan, were subjected to the *in vitro* examination (A1–3 and B1–4: Table 2). DNA was extracted from fruiting body tissues of the fungal specimens using the CTAB method, as described elsewhere [32]. PCR was conducted using the buffer system of Ampdirect Plus (Shimadzu) with BIOTAQ HS DNA Polymerase (Bioline) under a temperature profile of 95°C for 10 min, followed by 35 cycles at 94°C for 20 s, 47°C, 50°C, 53°C or 56°C for 30 s, and 72°C for 20 s (40 s for the entire ITS region), followed by 72°C for 7 min. The concentration of MgCl_2_, dNTPs, PCR primers and the template DNA in the reaction buffer were 1.5 mM, 200 µM, 0.5 µM and 1 ng/µl, respectively. Amplification of the DNA fragments was confirmed using the Flash Gel System for DNA (Lonza).

To evaluate the coverage of the selected fungal ITS primer pairs *in vitro*, an additional PCR assay was conducted under the optimal annealing temperature tested above using seven ascomycete and seven basidiomycete specimens (Table 2). All necessary permits of the sample collection were issued by Kyushu Regional Forest Office, Ministry of Agriculture, Forestry and Fisheries, Japan.

**Table 4:** Percentage of plant taxa amplified *in silico*.

			0 mismatch		1 mismatch		2 mismatches		3 mismatches	
Category	Subset	Target primername	Embryo-phyta	‘non-Embryo-phyta’	Embryo-phyta	‘non-Embryo-phyta’	Embryo-phyta	‘non-Embryo-phyta’	Embryo-phyta	‘non-Embryo-phyta’
SSU(forward)*	Subset 1 (64 Embryophytaand 252 ‘non-Embryophyta’LTUs)	NSA3	4.7	0.0	6.3	0.8	6.3	1.2	6.3	2.8
		ITS9mun	96.9	100.0	96.9	100.0	96.9	100.0	96.9	100.0
		NSI1	1.6	0.0	4.7	0.0	6.3	0.0	6.3	0.0
		ITS1-F	1.6	0.8	4.7	0.8	6.3	0.8	6.3	1.2
		ITS1-F_KYO1	4.7	0.8	6.3	0.8	6.3	0.8	6.3	1.2
		ITS1-F_KYO2	6.3	1.2	9.4	1.2	95.3	79.0	95.3	98.4
		ITS5	6.3	1.2	7.8	1.6	93.8	79.0	98.4	98.8
		ITS1	95.3	81.7	96.9	82.1	98.4	83.3	98.4	83.3
5.8S(reverse)†	Subset 2 (4,304 Embryophytaand 397 ‘non-Embryophyta’LTUs)	ITS2	5.9	1.0	94.3	19.6	97.2	19.6	97.6	25.2
		58A2R	6.1	4.3	93.2	12.8	95.2	12.8	95.5	13.4
		ITS10mun	6.0	4.3	92.2	12.8	95.1	12.8	95.4	13.4
		ITS2_KYO1	75.8	37.5	97.3	46.3	97.7	58.9	97.8	73.8
		ITS2_KYO2	77.0	37.3	98.3	51.9	98.8	61.5	98.9	66.2
5.8S(forward)‡	Subset 2 (4,304 Embryophytaand 397 ‘non-Embryophyta’LTUs)	ITS3	5.9	1.0	94.3	22.4	96.4	45.3	97.3	51.9
		58A1F	5.9	1.0	28.1	30.5	28.5	46.3	28.6	47.9
		58A2F	6.1	4.3	95.7	37.5	98.0	37.8	98.6	52.1
		ITS3_KYO1	75.8	37.5	98.3	37.8	98.6	55.7	98.7	71.0
		ITS3_KYO2	77.0	37.3	98.3	51.6	98.8	60.7	99.0	80.1
LSU(reverse)§	Subset 3 (130 Embryophytaand 47 ‘non-Embryophyta’LTUs)	ITS4	73.8	10.6	96.2	25.5	96.2	83.0	96.2	93.6
		ITS4_KYO1	73.8	10.6	96.2	25.5	96.2	83.0	96.2	93.6
		ITS4_KYO2	76.9	70.2	96.2	93.6	96.2	95.7	96.2	95.7
		ITS8mun	2.3	27.7	5.4	27.7	95.4	48.9	96.2	55.3
		ITS4_KYO3	1.5	38.3	3.1	48.9	20.8	66.0	94.6	89.4
		ITS4-B	0.0	0.0	0.0	0.0	0.0	0.0	0.0	0.0
		NLB4	1.5	2.1	1.5	2.1	14.6	12.8	16.2	23.4
		NLB3	1.5	2.1	1.5	2.1	14.6	14.9	16.2	53.2
		NLC2	1.5	2.1	3.1	17.0	3.8	51.1	3.8	53.2
		NL6Amun	0.0	0.0	0.8	0.0	0.8	0.0	2.3	2.1
		NL6Bmun	0.0	0.0	0.8	0.0	1.5	0.0	2.3	2.1

From zero to three nucleotide mismatches between each target primer and database sequences were allowed in ecoPCR, except for the two bases at the 3′-end of the primer.

* ITS2, which was used for the construction of subset 1, was used as a reverse primer in ecoPCR.

† ITS5, which was used for the construction of subset 2, was used as a forward primer in ecoPCR.

‡ ITS4, which was used for the construction of subset 2, was used as a reverse primer in ecoPCR.

§ ITS3, which was used for the construction of subset 3, was used as a forward primer in ecoPCR.

**Figure 3:**
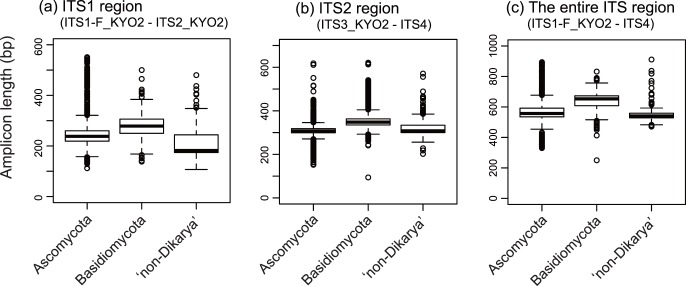
Length of sequence fragments amplified *in silico* by each primer pair. A box-and-whisker plot is shown for each primer set separately for Ascomycota, Basidiomycota, and “non-Dikarya” fungi. Median (bold line) and lower/upper quantiles are represented by a central box, and outliers outside the 1.5-fold range between lower/upper quantiles are indicated by circles. One mismatch to each target primer was allowed in the *in silico* PCR. (a) Length of sequence fragments amplified with ITS1-F_KYO2 and ITS2_KYO2 (ITS1 region). (b) Length of sequence fragments amplified with ITS3_KYO2 and ITS4 (ITS2 region). (c) Length of sequence fragments amplified with ITS1-F_KYO2 and ITS4 (the entire ITS region).

## Results

### *In silico* PCR and the Coverage of Fungal ITS Primers

Within the first group of ITS primers for the small subunit ribosomal RNA gene (Table 1, Fig. 1), ITS9mun, ITS1-F_KYO2, and ITS5 realized more than 98% coverage across 723 fungal LTUs when one mismatch between a target primer and the template sequence was allowed. Commonly used primers, such as ITS1-F and ITS1, showed less than 90% coverage (Table 3, Fig. 2). Among the reverse primers for the 5.8S ribosomal RNA gene (Table 1, Fig. 1), ITS2_KYO1 and ITS2_KYO2 showed more than 98% coverage under one-mismatch conditions, while the coverage of commonly used ITS2 primers was relatively low (93.7%; Table 3, Fig. 2). Likewise, among the forward primers for the 5.8S ribosomal RNA gene (Table 1, Fig. 1), 58A2F, ITS3_KYO1, and ITS3_KYO2 showed more than 98% coverage under one-mismatch conditions, while the coverage of commonly used ITS3 primers was relatively low (95.1%; Table 3, Fig. 2). Among the reverse primers for the large subunit ribosomal RNA gene (Table 1, Fig. 1), the widely used ITS4 showed taxon coverage as high as the newly designed ITS4_KYO1, ITS4_KYO2, and ITS4_KYO3 under one-mismatch conditions, while other primers showed much lower coverage (Table 3, Fig. 2). Among primers for the large subunit ribosomal RNA gene, ITS4_KYO3 showed low coverage across “non-Dikarya” fungi (77.8%), while others covered more than 90% of “non-Dikarya” taxa. *In silico* PCR also indicated that primers with more than 97% coverage across fungal LTUs (i.e., ITS9mun, ITS1-F_KYO2, ITS5, ITS2_KYO1, ITS2_KYO2, 58A2F, ITS3_KYO1, ITS3_KYO2, ITS4, ITS4_KYO1, and ITS4_KYO2) are unlikely to exhibit a significant coverage bias toward ascomycetes or basidiomycetes (Table 3). Although a relatively small number of “non-Dikarya” sequences was available, a further inspection of sequences in the present data sets revealed that ITS1-F_KYO1, ITS1-F_KYO2, ITS2_KYO2, ITS3_KYO2, and ITS4 matched 99.2% (123/124), 96.0% (119/124), 98.8% (568/575), 99.0% (569/575), and 99.2% (387/390), respectively, of glomeromycetous sequences (one-mismatch conditions).

**Figure 4:**
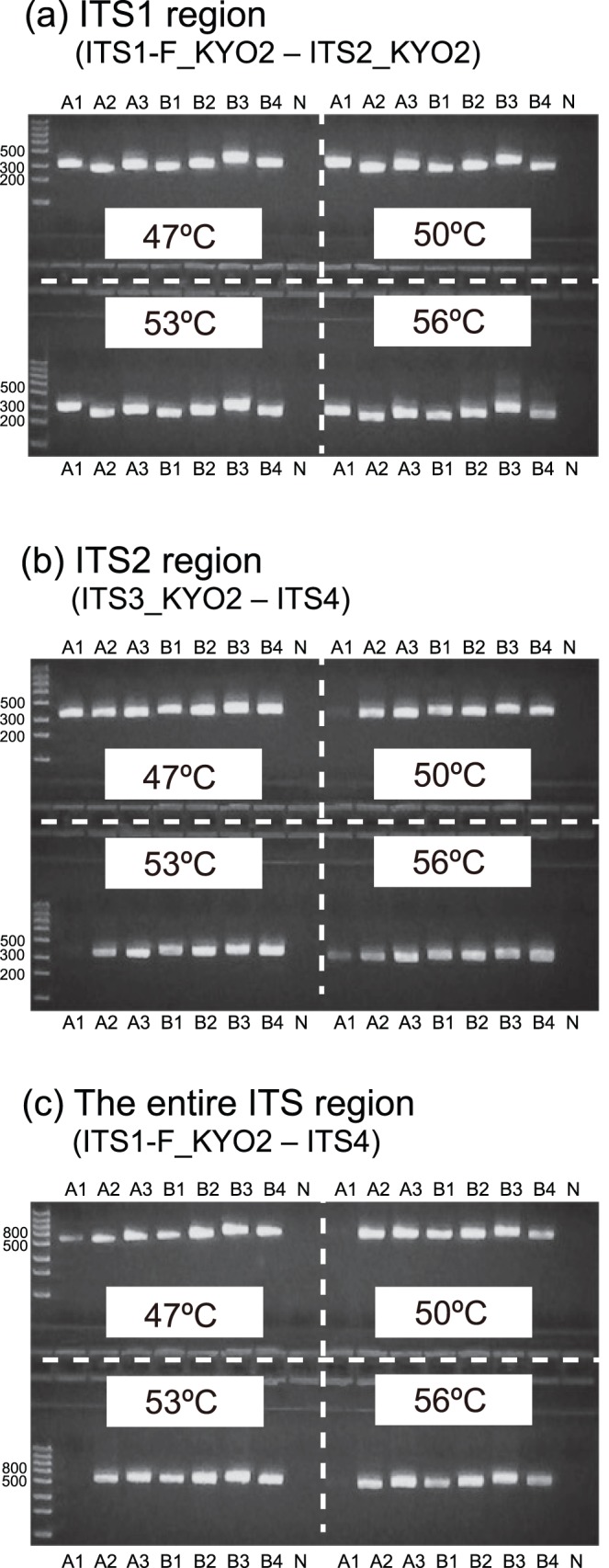
Exploration of annealing temperatures. Three ascomycete and four basidiomycete species were subjected to amplification of the (a) ITS1 region (ITS1-F_KYO–ITS2_KYO2), (b) ITS2 region (ITS3_KYO2–ITS4), and the (c) entire ITS region (ITS1-F_KYO2–ITS4) at four annealing temperatures (electrophoresed on 2.2% agarose gels). See Table 2 for the abbreviations of fungal specimens. N, negative control.

### Mismatches to Plant ITS Sequences

Among the above-mentioned high-coverage fungal primers, ITS1-F_KYO2 and ITS5 showed less than 10% coverage of plant LTUs under one-mismatch conditions, while other high-coverage primers matched the sequences of more than 96% of plant LTUs (Table 4). Thus, although ITS9mun showed the highest coverage across fungal LTUs (Table 3, Fig. 2), potential simultaneous amplification of plant sequences by this primer would be unfavorable in a variety of contexts, such as DNA barcoding of mycorrhizal fungi. Meanwhile, ITS1-F_KYO1, whose LTU coverage was relatively high (96.8%), showed a high specificity for fungi (Table 4). At the large subunit ribosomal RNA gene, ITS4_KYO3 matched the sequences of only 3.1% of land plant LTUs, while other primers were expected to amplify most land plant LTU sequences (Table 4). In terms of the 5.8S RNA gene, no primer with more than 95% fungal LTU coverage under one-mismatch conditions was expected to selectively amplify fungal sequences (Table 4). Accordingly, because of coverage across fungal LTUs and mismatches to plant ITS sequences, ITS1-F_KYO2–ITS2_KYO2, ITS3_KYO2–ITS4, and ITS1-F_KYO2–ITS4 primer pairs were tentatively selected as preferable for amplification of ITS1, ITS2, and the entire ITS regions, respectively, in the subsequent *in vitro* analyses (see below for further discussion). The average lengths of fragments amplified were 252.1 bp (SD = 53.2; *N* = 8,555 sequences) for the ITS1-F_KYO2–ITS2_KYO2 pair, 327.2 bp (SD = 40.1; *N* = 19,646) for the ITS3_KYO2–ITS4 pair, and 586.5 bp (SD = 73.9; *N* = 3,774) for the ITS1-F_KYO2–ITS4 pair (Fig. 3). Although the basidiomycete amplicons were the longest on average, considerable variation in amplicon lengths was observed within the ascomycete, basidiomycete, or “non-Dikarya” group (Fig. 3).

**Figure 5:**
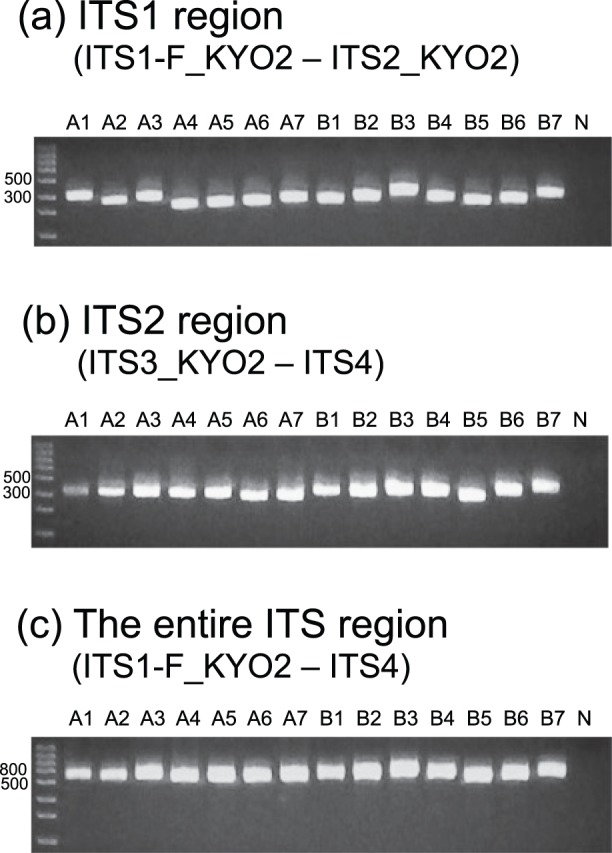
Taxon coverage of respective primer pairs. Seven ascomycete and seven basidioimycete species were subjected to amplification of the (a) ITS1 region (ITS1-F_KYO–ITS2_KYO2), (b) ITS2 region (ITS3_KYO2–ITS4), and the (c) entire ITS region (ITS1-F_KYO2–ITS4) at 47°C (electrophoresed on 2.2% agarose gels). See Table 2 for the abbreviations of fungal specimens. N, negative control.

### *In vitro* Selection of Annealing Temperatures

*In vitro* PCR analysis revealed that the optimal annealing temperature was 47°C for all the primer pairs examined (Fig. 4). The seven ascomycete and seven basidiomycete fungi examined were successfully PCR-amplified using the primer pairs (Fig. 5).

## Discussion

Using nucleotide sequence databases, we designed improved fungal ITS primers for the DNA barcoding of fungi. Our *in silico* PCR analysis indicated that the coverage of the newly designed primers was around 99% (e.g., 98.8% for ITS1-F_KYO2, 99.1% for ITS2_KYO2, and 99.2% for ITS3_KYO2), and the taxonomic bias of amplification (ascomycetes vs. basidiomycetes) was expected to be very low (Table 3, Fig. 2). Concomitantly, *in vitro* analyses demonstrated that the improved primers are applicable to the cataloging and identification of ascomycete and basidiomycete fungi using standard PCR conditions (Figs. 4 and 5).

### Requirements for Fungal DNA Barcoding Primers

In fungal DNA barcoding founded on PCR-based next-generation sequencing of environmental samples such as soils, roots, and leaves, three important criteria exist regarding primer selection. First, as a general requirement for primers used in the exploration of biodiversity, fungal DNA barcoding primers should amplify sequences of broad fungal taxa to accurately describe the diversity and community structures of fungal species in environmental samples. As reported recently, several classic primers widely used in DNA barcoding of fungi (e.g., ITS1, ITS1-F, ITS2, and ITS3) do not cover a non-negligible proportion of fungal taxa (Table 3, Fig. 2), potentially causing taxonomic bias in the data [39]. Meanwhile, the newly designed primers ITS1-F_KYO2, ITS2_KYO2, and ITS3_KYO2 are expected to match the sequences of *c*. 99% of fungal LTUs, thereby enabling thorough and unbiased DNA barcoding of ascomycete and basidiomycete fungi. Results also showed that three classic primers, ITS9mun, ITS5, and ITS4, exhibited broad coverage of fungal LTUs (Table 3, Fig. 2). In particular, the coverage of ITS4 was as broad as that of any other primer, including our newly designed primers (Table 3, Fig. 2), suggesting that the classic primer is appropriate for fungal DNA barcoding as well as the newly-designed primers targeting the 5′-end of large subunit ribosomal RNA gene. Given the relatively small number of “non-Dikarya” sequences in the public databases, the coverage of the newly designed primers of fungal groups other than ascomycetes and basidiomycetes remains to be evaluated. Nonetheless, inspection of sequences in the present data sets revealed that concerning the “non-Dikarya” mycorrhizal fungal phylum Glomeromycota, ITS1-F_KYO1, ITS1-F_KYO2, ITS2_KYO2, ITS3_KYO2, and ITS4 matched with 96.0–99.2% of the sequences examined *in silico*. Thus, these primers are useful for not only DNA barcoding of ascomycetes and basidiomycetes, but also cataloging of glomeromycetes (and other “non-Dikarya” fungi) in the environment. In fact, pyrosequencing with the newly-designed primers successfully yielded glomeromycete ITS sequences in metagenomic analyses of root-tip and soil samples collected in Japanese temperate forests (Toju et al., unpubl.).

The second important criterion in selection of fungal barcoding primers is selective amplification of fungal sequences, i.e., the elimination of plant and other organismal sequences in PCR of mycorrhizal, leaf, or soil samples. Some fungi-specific primers such as ITS1-F and ITS4-B are widely used in DNA barcoding of various environmental samples (reviewed in [23]), although their low coverage of fungal taxa is expected to preclude comprehensive analyses of fungal diversity and community structures [39] (Table 3, Fig. 2). Basically, a trade-off exists between specificity for fungi (e.g., exclusion of plant taxa) and unbiased amplification within the fungal kingdom. Due to this trade-off, barcoding primer sets should be selected based on the purpose of the ecological/microbiological study. As substitutes for the classic fungi-specific primer ITS1-F, ITS1-F_KYO1 and ITS1-F_KYO2 were designed for selective amplification of fungal ITS sequences. *In silico* PCR showed that ITS1-F_KYO1 showed a high specificity for fungi that was comparable to that of the widely used primer ITS1-F; in contrast, ITS1-F_KYO2 might amplify sequences of diverse plant taxa under relaxed PCR conditions (e.g., low annealing temperatures). Thus, ITS1-F_KYO1 can be used as a highly specific primer for fungi at the slight expense of universality, while ITS1-F_KYO2 is a high-coverage primer, whose risk of amplifying plant sequences should be mitigated by adopting relatively stringent PCR conditions. Potential matches between these fungus-specific primers and other soil eukaryotic sequences (e.g., nematodes, springtails, and protozoa) warrants further extensive *in silico* and *in vitro* analyses.

The third criterion for the selection of barcoding primers is amplicon length. Next-generation sequencing is an invaluable technique for high-throughput DNA barcoding of fungi, being applied particularly to metagenomic analyses of mycorrhizal, phyllosphere, and soil fungi [9], [37], [50]. However, output read length is shorter in most next-generation sequencers than in Sanger sequencers (e.g., Applied Biosystems 3130 Genetic Analyzer), which has precluded DNA barcoding of fungi based on the entire ITS region. For example, since the average read length obtained from 454 pyrosequencing has until recently been 250 bp, pioneering studies on fungal metagenomics usually target the ITS1 region for DNA barcoding [9], [46], [51], [52]. The release of the GS FLX Titanium series (454 Life Sciences), which extended the length of output sequences to 400 bp, has allowed use of the potentially more informative ITS2 region for DNA barcoding [50], [53]. Given that the fungal ITS2 region can be highly variable at the species level [54], primers for amplification of this region should enable identification of fungal species and subsequent analyses of fungal communities [50], [53]. Therefore, DNA barcoding of the ITS2 region using ITS3_KYO2 and ITS4 with sequencers whose read length is less than 700 bp would be sufficiently informative in the context of ecological and microbiological studies (Fig. 3). Meanwhile, DNA barcoding targeting the entire ITS region using ITS1-F_KYO2 and ITS4 will increase the reliability of species identification if the upgraded FLX Titanium, whose read length is up to 1,000 bp, is used.

### Future Directions

We herein developed high-coverage fungal ITS primers, which facilitate investigation of the remarkable diversity of fungi. Some of the newly designed primers enable selective amplification of fungal ITS sequences and are useful for investigating fungal diversity and community structures within mycorrhizal associations, leaf, soil, and other environmental samples that potentially contain plant tissues and detritus. There can be little doubt that DNA barcoding will dramatically enhance our knowledge of fungal diversity and communities during the next decade. To further fuel use of fungal DNA barcoding in ecological and microbiological studies, the potential bias caused by primers should be quantitatively evaluated based on next-generation sequencing [46].

Although the number of ITS sequences in public sequence databases is growing rapidly [22], [23], many studies use small or large subunit ribosomal RNA sequences as a DNA barcoding marker [34]–[38]. ITS sequences are hypervariable in most fungal clades and are used particularly in the context of identification at species or lower levels [25], while the considerable variation in the length of this region often impedes multiple sequence alignments and subsequent phylogenetic analyses. In contrast, small or large subunit sequences are more conserved across the fungal kingdom than ITS sequences, enabling, e.g., phylogenetic investigation of novel fungal clades. Thus, the simultaneous use of ITS and small/large subunit regions is desirable in future studies based on DNA barcoding after the read length of high-throughput sequencers is extended. To simultaneously conduct identification at species or lower levels and phylogenetic analyses, the ITS primers we report here should be paired with primers for amplification of small or large subunit ribosomal RNA sequences.
